# A study on classification based concurrent API calls and optimal model combination for tool augmented LLMs for AI agent

**DOI:** 10.1038/s41598-025-06469-w

**Published:** 2025-07-01

**Authors:** HeounMo Go, SangHyun Park

**Affiliations:** Department of Computer Science, Yonsei University, Yonsei Univ, Yonsei‑ro 50, Seodaemun‑gu, Seoul, 03722 Republic of Korea

**Keywords:** Computer science, Information technology

## Abstract

AI Agents have evolved to not only recommend content but also facilitate information retrieval and task processing. Developing AI Agents using general-purpose LLM models necessitates integration with external tools, leading to tool-augmented LLM studies. Despite the availability of multiple tools for the same purpose, existing research has not fully leveraged this diversity. This study categorizes external tools by type and proposes a method to simultaneously call tools of the same type. This allows for the utilization of diverse external tools in LLM inference, thereby achieving a higher accuracy compared to when only a single tool for one task is used. Experimental results show an accuracy improvement of 4.4–9.3% over existing studies. Furthermore, when utilizing tool-augmented LLM, a multi-step reasoning approach that divides the process into stages such as planning and tool invocation is widely employed. With the rapid advancement of LLMs, enhanced models continue to emerge. Considering the trade-offs between performance and cost in models, it is crucial to find an optimal combination of models in each stage of tool augmented LLM. In this study, we propose a novel method for efficiently utilizing both enhanced LLM models and existing models, which reduces response errors by up to 9%.

**Supplementary Information:**

The online version contains supplementary material available at 10.1038/s41598-025-06469-w.

## Introduction

The success of ChatGPT has heightened interest in natural language-based AI Agents. AI Agents have evolved to handle tasks such as personalized content recommendations, information retrieval, action completion including restaurant reservations, and product purchases through familiar conversational interfaces. Consequently, many companies are leveraging AI Agents to enhance their business competitiveness. For instance, SK Telecom offers the AI Agent service, “A.,” to enable customers to use artificial intelligence more easily and conveniently in their daily lives. A. provides a “Daily” feature that integrates personal tasks such as to-dos, schedules, and records, allowing users to manage calendars, tasks, routines, and sleep in a unified manner. This service supports users in managing their daily activities effortlessly by allowing them to communicate with A. as if speaking to a personal assistant, thereby storing and managing appointments, meetings, and tasks without the hassle of manual input.

The AI assistant experience is significantly enhanced by offering personalized suggestions that consider various factors such as weather, traffic, and the user’s preferences and tastes when performing schedules and appointments. Additionally, A. provides a natural conversational experience based on a large language model and offers a variety of agent-based services, including music, media, stock trading, and movie reservations. Through the multi-LLM agent, users can simultaneously receive and compare responses from seven of the latest LLMs, including ChatGPT, Perplexity, Claude, and A.X. Microsoft has integrated MS Copilot into its Office suite to boost productivity in document tasks, and AI Agents are being actively developed to improve the efficiency of call centers, helping and potentially replacing human agents. AI Agents are also being explored for productivity and cost reduction in fields like marketing and coding.

These AI Agents are built on LLM models, which can be proprietary, or external models like ChatGPT given the high costs of developing and maintaining proprietary LLMs. While general-purpose LLM models like ChatGPT perform well in open-domain Q&A due to extensive training data, integrating with various external tools is essential for real-world applications involving domain-specific knowledge and task execution, which is called tool augmented LLM. For example, an AI Agent must integrate with a weather information API to provide local weather forecasts or with a booking API to handle accommodation reservations.

Typically, there are several kinds of tools even for same purpose. For example, google search API and Bing web search API are available for information search, and there are multiple weather forecast APIs in each country. However, existing research has not fully leveraged this diversity for more accurate responses. This study categorizes external tools by type for real-world AI Agent applications and proposes a method to simultaneously call tools of the same type, inputting their results into the LLM model to enhance inference accuracy. This allows for the utilization of the execution results from a more diverse range of external tools in LLM inference, thereby achieving a higher response accuracy compared to when only a single tool for one task is used. Furthermore, when utilizing tool-augmented LLM, a multi-step reasoning approach that divides the process into stages such as planning and tool invocation is widely employed. With the rapid advancement of LLMs, increasingly enhanced models continue to emerge. However, high-performance models require significant token costs, making it crucial to find an optimal combination of models that considers both performance and cost. In this study, we propose a novel method for efficiently combining and utilizing both enhanced LLM models and existing models.

## Related work

While LLMs demonstrate high performance across various domains, research on tool augmented LLM, which is integrating external tools with LLM, has been active for enhancing reasoning performance and accuracy.

Initial studies focused on enhancing LLM inference capabilities to improve responses to common-sense questions, mathematical queries, and symbolic reasoning^1^. Research has also explored using external tools to enhance natural language processing and image recognition performance, such as Tool Former^2^ and Visual GPT^3^. Additionally, studies like ReAct^4^, ART^5^, MM-ReAct^6^, TaskMatrix.AI^7^, and ReWOO^8^ have investigated multi-step reasoning and external tool integration to improve problem-solving capabilities.

Addressing the hallucination problem, where LLMs provide incorrect answers in domain-specific knowledge tasks, has also been a focus^9–11^. Solutions include integrating external tools during action execution^12–16^, with particular attention to real-world API utilization^17–21^. Research has explored various APIs to enhance LLM performance, such as search APIs^4,22,23^, calculator APIs for error correction^24^, code interpreter APIs for code generation quality^25^, and math prover APIs for mathematical problem proofs^26^.

Typically, there are several kinds of tools even for a same purpose. For example, google search API and Bing web search API are available for information search, and there are multiple weather forecast APIs in each country. Despite the availability of multiple APIs for the same purpose, existing studies have not fully leveraged this diversity for more accurate responses, which means that if we allow for the utilization of the execution results from a more diverse range of external tools in LLM inference, we can achieve a higher response accuracy compared to when only a single tool for one task is used as previous studies do. This study categorizes external APIs by purpose and type for real-world AI Agent applications and proposes a method to simultaneously call APIs of the same type, inputting their results into the LLM model to enhance inference accuracy. This process includes exception handling to minimize unnecessary token costs.

Additionally, as LLM models continue to evolve, there are various versions of models available even in a same type of LLM, differentiated by performance and price. From the perspective of companies developing real world AI agent services, it is necessary to efficiently combine these various models according to the purpose and characteristics of the service. This study proposes a method to efficiently combine new and high-performance models with existing models when using external tools.

## Methods

### Work flow

This study uses ReWOO^8^ as the base methodology. ReWOO enhances performance by utilizing external tools through multiple inference steps and establishes an overall inference plan in a single LLM call during the Plan stage, avoiding multiple steps like CoT^27^ and ReAct^4^.

**Fig. 1:**
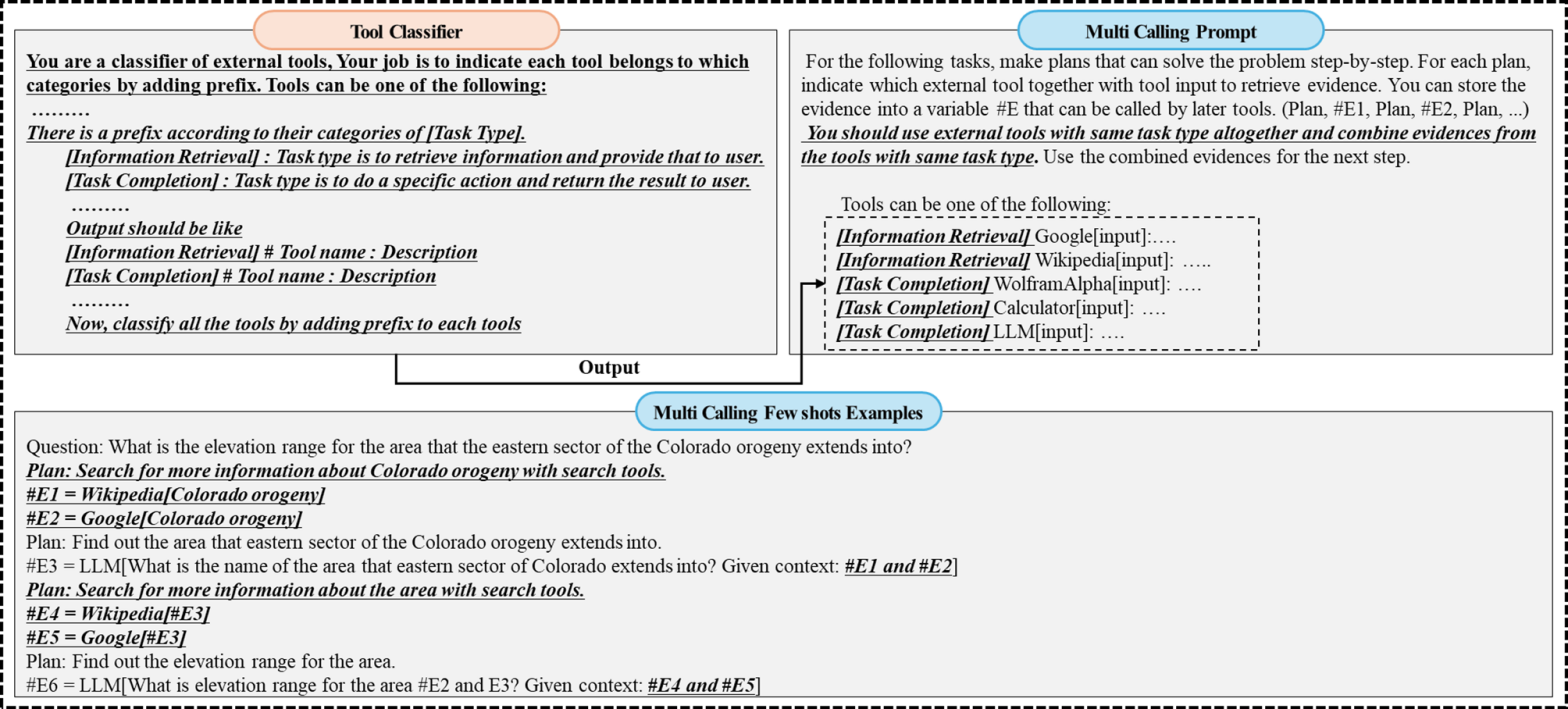
Planner Workflow – Tool Classifier designed to categorize external tools based on their specific purposes, Multi Calling Prompt and Few shot examples that allows for the simultaneous invocation of these tools.

The workflow consists of three main stages: Planner, Worker, and Solver. The Planner stage establishes an overall plan to solve the given problem, as illustrated in Fig. 1.

The first step in the Planner stage is to classify the given external tools by purpose using a Tool Classifier, which is done through a prompt to the LLM. The Tool Classifier serves the function of categorizing external tools into different types based on their purpose-specific similarities, by adding a prefix to the tool description. To achieve this, it provides the Large Language Model (LLM) with a prompt that includes candidate types, their descriptions, and examples of the output format with the prefix. Figure 1 shows an example. Here, there are two categories: Information Retrieval and Task Completion. The reason why we choose these two categories as an example is described in the following section.

Multi Calling Prompt calls tools of the same classification simultaneously with the categorization prefix from Tool Classifier. Few-shot examples also call tools of the same classification simultaneously, passing their results to the next stage. Figure 1 shows an example where Wiki and Google tools, classified for Information Retrieval, are called simultaneously, passing both results to the next stage. Calling multiple tools with the same purpose simultaneously increases response accuracy by utilizing results from other tools if one tool fails to provide the desired outcome. The Tool Classifier and Multi Calling Prompt for simultaneous tool calls are key contributions of this study, highlighted in bold and underlined in Fig. 1.

In the next stage, the Worker calls the actual external tools according to the plan established by the Planner, retrieving evidence for each step. If the external tool’s result is deemed unsatisfactory, exception handling is used to prevent unnecessary tokens from being input into the LLM, which is a contribution of this study. For example, if a search tool’s result includes “not find,” it is replaced with null to avoid unnecessary token costs.

Finally, the Solver inputs the collected evidence into the LLM according to the plan to derive the answer to the problem.

### Classification criteria

For real-world applications, optimizing the classification of external tools by purpose is necessary. A broader classification may result in calling too many external tools simultaneously, potentially exceeding the LLM’s context window and increasing token costs. Conversely, a narrower classification may diminish the effect of calling multiple tools simultaneously.

This study verifies the effectiveness of the proposed method by considering two types: “Information Retrieval” for widely used search tools like Google and Wiki, and “Task Completion” for specific tasks like arithmetic operations. The two classification types presented in this study as examples are the most commonly used in AI Agent service use cases. For the most representative AI Agent, ChatGPT, the most typical use case is providing information retrieval results in a question-and-answer format. Recently, there has been an effort to expand its capabilities towards task completion through features such as function calls. Although this study only applies classification by purpose, domain-specific classifications could be added for real-world applications. For example, domains like “Contents” for music and videos, or “Accommodation” and “Airline” for travel reservations, and “Restaurant” for dining recommendations. If domain classifications are added, the Tool Classifier’s input prompt and examples can be modified to include two-step prefixes, such as [Information Retrieval] [Contents], and included in the Multi Calling Prompt. This will be addressed in future work.

### LLM combination optimization

With the rapid advancement of LLM models, more evolved models continue to emerge. For instance, ChatGPT has released versions like 3.5 turbo, 4o, 4o-mini, and o1. While newer models generally offer better performance, they also tend to have higher token costs. Although the latest models would be applied for performance, it would require more token cost.

In addition, considering the high degree of freedom in the responses of Large Language Models, the adoption of new models could significantly impact the service, which means that companies should consider additional verification and operational costs when switching from optimized services based on existing models. Considering these aspects, it is necessary to efficiently combine the existing model with the new model rather than completely replacing it with a new LLM all at once.

This study investigates the most efficient combination of LLM models for the Planner and Solver stages, using the widely used ChatGPT 3.5 turbo model and the advanced GPT 4o model. The reason for selecting the ChatGPT 3.5 turbo model and the GPT 4o model for this experiment is that the ChatGPT 3.5 turbo is one of the oldest and most affordable models provided by OpenAI, whereas the GPT 4o is one of the most expensive and latest models. This study aims to analyze the performance variation trends between a combination of a low-cost, older model and a high-cost, latest model, which is why these two models were chosen.

## Experiment

### Datasets

The datasets used in this study are listed in Table 1.

**Table 1:** Tasks and datasets.

Task	Dataset	Description
Common knowledge and reasoning	HotpotQA ^28^	Multi-hop reasoning QA task over diversified domains
	StrategyQA^29^	Open domain QA task where answers require steps of reasoning.
	Sports Understanding^30^	Factual QA benchmark from BigBench[32] over sports domain
Scientific reasoning	PhysicsQuestion^31^	High school physics questions.
Curated	SOTUQA^8^	QA dataset over State of the Union Address 2023

In selecting the dataset used for this study, the following factors were considered. First, we chose publicly available data widely used in the commonsense and reasoning domains of open domain applications where Large Language Models (LLMs) are extensively utilized (Hotpot QA and Strategy QA). Specifically, we aimed to select datasets that could verify multi-hop reasoning abilities. (Hotpot QA) To test response capabilities in specific domains outside the open domain, we included the Sports Understanding data. Additionally, to verify response capabilities for different types of tasks, such as arithmetic calculations rather than general reasoning or question-answering, we incorporated the Physics Question data. Lastly, to assess the performance of the proposed method on commonly used curated data, we aimed to evaluate its performance using the SOTUQA data.

### Baselines

To verify the effectiveness of the proposed approach, comparisons is made with the Direct method, which inputs questions directly into the LLM without step-by-step prompts, and ReWOO^8^, one of the latest multi-step reasoning methods using external tools.

### Models

The gpt-3.5-turbo-1106 model, widely used in LLM-based services, is primarily used, and the gpt-4o-2024-05-13 model is used to find the optimal combination.

### External tools

The external tools used in this study are listed in Table 2.

**Table 2:** External tools.

Type (prefix)	Tool	Description
Information Retrieval	Wikipedia	Search engine for Wikipedia
	Google	Search result from Google SERP API
Task Completion	WolframAlpha	Computation result from Wolfram Alpha API
	Calculator	Program-aided LLM
	LLM	Separate single LLM

### Metrics

Exact match (EM) and character-level F1 scores are used for performance comparisons, which are widely used in natural language processing. Additionally, accuracy is calculated through GPT-based answer comparisons to measure semantic accuracy, which is the main metric for evaluation. Efficiency is compared by calculating the number of tokens and costs used by the planner, worker, solver, and external tools, as well as the number of inference steps. For inference steps, the direct method is counted as 1, and the proposed method and ReWOO are counted as the number of steps proposed by the Planner + 1, considering the Solver stage as a single step.

## Results

### Comparison with baselines

**Fig. 2:**
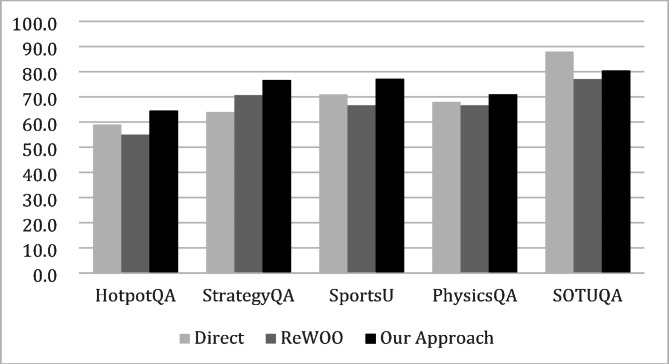
Comparison with baselines.

Table 3 shows that the proposed method generally achieves higher accuracy than the comparison methods, as illustrated in Fig. 2.

**Table 3:**
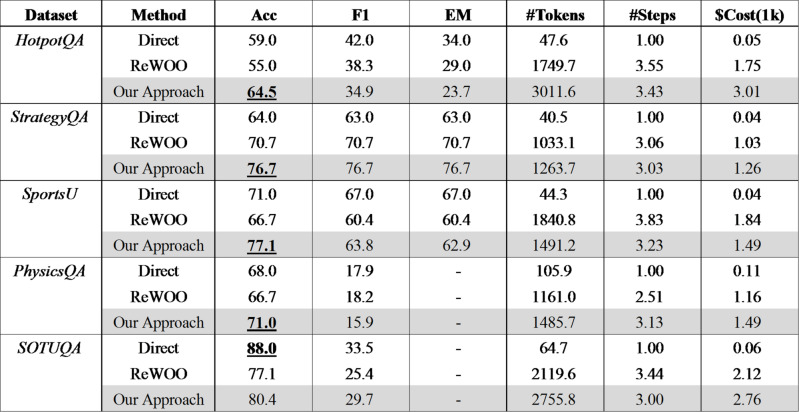
Comparison with baselines.

Compared to the highest-performing baseline, the proposed method shows an accuracy improvement of + 9.3% for HotpotQA, + 8.5% for StrategyQA, + 8.7% for Sports Understanding, and + 4.4% for PhysicsQA. Character-level F1 and Exact Match show inconsistent trends, indicating that word-level comparisons alone are insufficient for measuring accuracy in complex questions. Although token count and cost are higher than ReWOO due to simultaneous tool calls, the variation is dataset-dependent, with Sports Understanding showing a lower token count. For complex problems like HotpotQA, simultaneous tool calls result in more tokens, leading to higher token increases. However, for simpler datasets like StrategyQA and PhysicsQA, token increases are relatively lower, 22% and 28% respectively. Interestingly, Sports Understanding shows a 19% decrease in token count, likely due to filtering external tool results such as nullifying “not find” results, minimizing unnecessary token costs. This suggests that appropriate filtering can minimize token cost increases even with simultaneous tool calls.

The number of steps is similar to ReWOO. Overall, the proposed method shows accuracy improvements and potential for minimizing token cost increases with proper filtering. In the case of the number of steps, as expected, the direct approach shows a count of 1, while both ReWOO and our approach exhibit a similar count of approximately 3. The reason for the number of steps not increasing in our approach is presumed to be due to the handling of simultaneous calls to the same type of API in a single step, rather than treating them as separate steps.

For SOTUQA, the direct LLM call without external tools shows the highest accuracy, likely due to the LLM model’s continuous updates incorporating relevant training data.

In summary, the experiment result demonstrates an improvement in accuracy ranging from 4.4 to 9.3% compared to existing studies, based on experimental results. Although the token cost increased by 22% (Strategy QA) to 72% (Hotpot QA) with additional API usage comparing with baseline methods, the token cost in domain specific dataset (Sports Understanding) shows a reduction of up to 19% through filtering. This suggests that more precise filtering of results from multiple API calls, or even the use of a dedicated Small Language Model (SLM) for this purpose, could further reduce token costs. The proposed methodology not only shows meaningful performance improvement but also indicates the potential for token cost optimization which will be addressed in future research.

### Model combination optimization

The accuracy and efficiency of different ChatGPT model combinations for the Planner and Solver stages for each dataset are compared in Table 4.

**Table 4:**
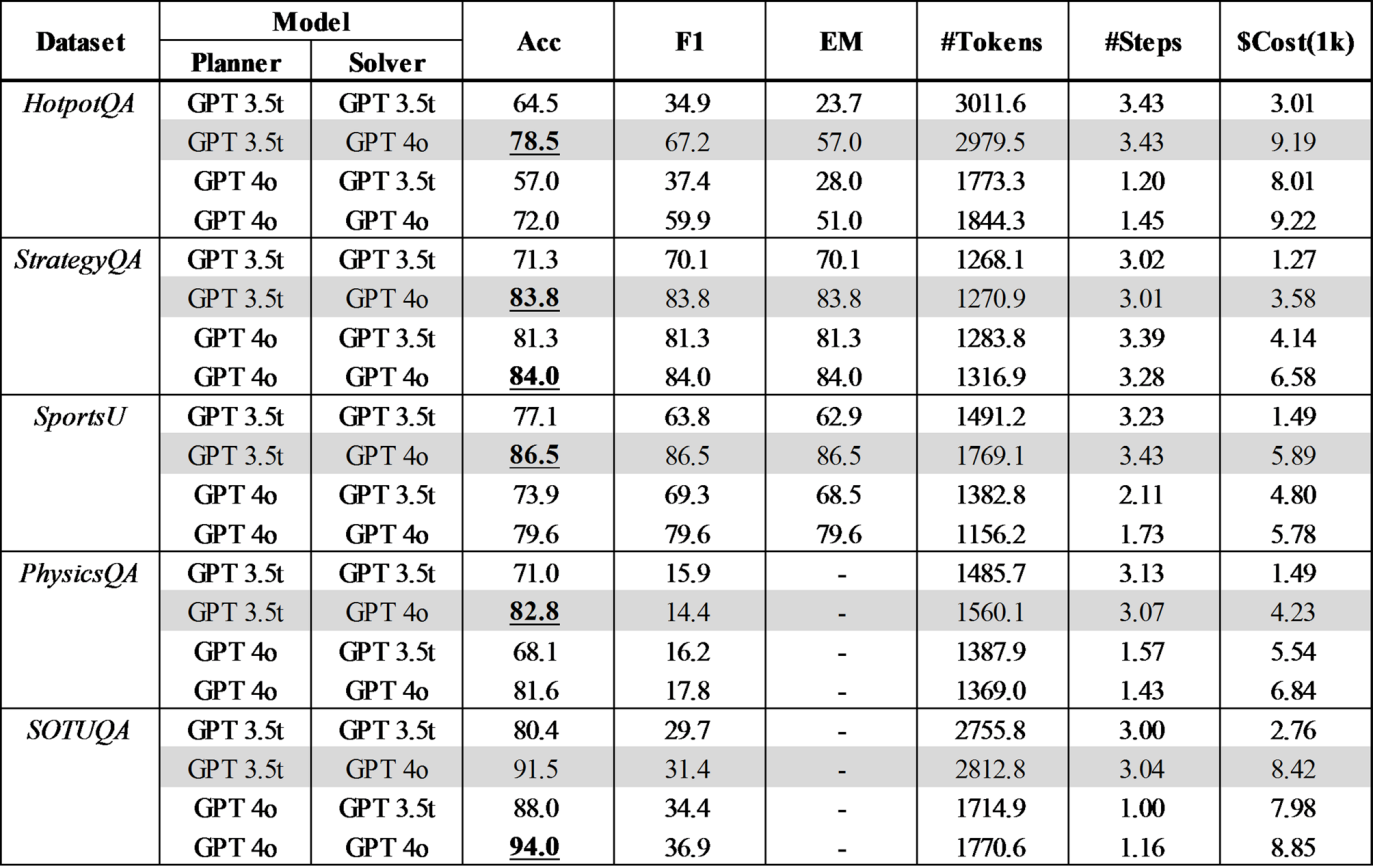
Model combination Optimization - Comparison between models of planner and Solver.

**Fig. 3:**
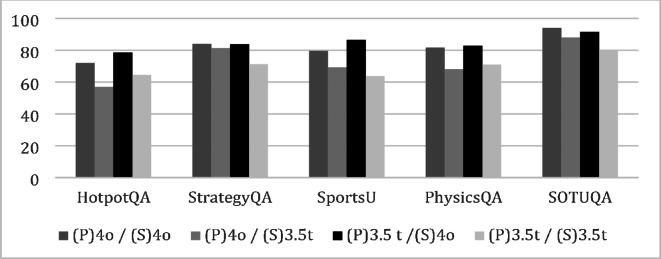
Planner and Solver Model Comparison.

GPT 3.5t means gpt-3.5-turbo-1106, and GPT 4o means gpt-4o-2024-05-13 model which is more advanced model. Figure 3 illustrates the accuracy results, with each case representing the Planner/Solver model combination.

Contrary to expectations, using GPT 3.5t for the Planner and GPT 4o for the Solver generally yields the highest accuracy. Compared to the highest-performing baseline, this combination shows an accuracy improvement of + 9.0% for HotpotQA, + 3.0% for StrategyQA, + 8.7% for Sports Understanding, and + 1.4% for PhysicsQA. F1 and EM are inconsistent, indicating that word-level comparisons are insufficient for measuring accuracy. Given the significant token cost difference between GPT 3.5t and GPT 4o, token costs rather than token counts should be considered for measuring cost efficiency.

For HotpotQA and Sports Understanding, the total token cost of GPT 3.5t/GPT 4o is similar to the cost of GPT 4o/GPT 4o. Meanwhile, for StrategyQA and PhysicsQA, GPT 3.5t/GPT 4o shows 46% and 38% token cost savings compared to using GPT 4o/GPT 4o respectively. Overall, compared to using GPT 4o for both stages, the proposed combination of GPT 3.5t/GPT 4o shows similar costs with 6.5% and 6.9% higher accuracy for HotpotQA and Sports Understanding respectively, and similar accuracy with 46% and 38% token cost savings for StrategyQA and PhysicsQA. For SOTUQA, the Planner using GPT 3.5t and Solver using GPT 4o shows similar accuracy and costs to using GPT 4o for both stages, likely due to the LLM model’s continuous updates incorporating relevant training data, making the Solver model’s performance more critical.

To analyze why the Planner using GPT 3.5t and Solver using GPT 4o yields the highest performance, examples of plans generated by each model are included in the Appendix. The GPT 4o plan, unlike the few-shot example, applies the Wiki and Google tools for different purposes, likely due to the model’s advanced reasoning capabilities, and it results in incorrect answers. The GPT 3.5t plan follows the few-shot example format, yielding correct answers. This suggests that higher LLM model sophistication does not always guarantee better performance, and in cases requiring more controllability than reasoning performance, existing models may be more suitable.

When using GPT-4o as the Planner, the number of steps is approximately 1 to 2, whereas using GPT-3.5t results in about 3 steps. This indicates that the number of steps is significantly smaller when GPT-4o is employed as the Planner. As previously mentioned, GPT-4o tends to generate plans that differ from the few-shot examples due to its enhanced inference capabilities, which is presumed to result in fewer steps.

## Conclusion

This study proposes a method to enhance the accuracy of AI Agent inference results by categorizing external tools by type and simultaneously calling tools of the same type, which allows for the utilization of the execution results from a more diverse range of external tools in LLM inference, thereby achieving a higher response accuracy compared to when only a single tool for one task is used. Experimental results show an accuracy improvement of 4.4–9.3% over existing studies. Furthermore, when utilizing tool-augmented LLM with multi-step reasoning approach, it is crucial to find an optimal combination of models in each step. We propose a new method combining enhanced LLM models with existing models efficiently reduces response errors by up to 9%. Future work will explore performance improvements through diversified classification criteria and domain-specific standards for real-world applications. This study can be utilized for various AI agents for open domain question & answering, new drug development and so on.

## Electronic supplementary material

Below is the link to the electronic supplementary material.

Supplementary Material 1

## Data Availability

Data and code for this study are available at https://github.com/ilyhs79/Concurrent-API-Calls-and-Optimal-Model-Combination-for-Tool-Augmented-LLMs.
