# Supplementary material for: A study on classification based concurrent API calls and optimal model combination for tool augmented LLMs for AI agent
Source: Sci Rep. 2025 Jul 1;15:20579. doi: 10.1038/s41598-025-06469-w (PMC12216082; doi:10.1038/s41598-025-06469-w)
Supplement: Supplementary file 1 — Supplementary Material 1 [file 41598_2025_6469_MOESM1_ESM.docx]

# **Supplementary information**

1. An example of plan by GPT 4o and solver result by GPT 4o

=== Planner ===

Begin! Describe your plans with rich details. Each Plan should be followed by only one #E.

The 1994–95 U.C. Sampdoria season included a midfielder who was of what nationality?

*****Plan: Search for more information about the 1994–95 U.C. Sampdoria season with search tools.*****

***#E1 = Wikipedia[1994–95 U.C. Sampdoria season]***

**Plan: Identify the midfielders from the 1994–95 U.C. Sampdoria squad.**

#E2 = LLM[Who were the midfielders in the 1994–95 U.C. Sampdoria squad? Given context: #E1]

****Plan: Search for more information about the nationalities of the identified midfielders.****

**#E3 = Google[Nationality of midfielder {Midfielder Name from #E2} 1994–95 U.C. Sampdoria]**

**Plan: Determine the nationality of the identified midfielder(s).**

#E4 = LLM[What was the nationality of the midfielder(s) from the 1994–95 U.C. Sampdoria squad? Given context: #E3]

Begin! Describe your plans with rich details. Each Plan should be followed by only one #E.

=== Solver ===

Solve the following task or problem. To assist you, we provide some plans and corresponding evidences that might be helpful. Notice that some of these information contain noise so you should trust them with caution.

The 1994–95 U.C. Sampdoria season included a midfielder who was of what nationality?

Now begin to solve the task or problem. Respond with the answer directly with no extra words.

The 1994–95 U.C. Sampdoria season included a midfielder who was of what nationality?

Brazilian

Q: The 1994–95 U.C. Sampdoria season included a midfielder who was of what nationality?

Truth: English

Answer: Brazilian

***Judgement: INCORRECT***

1. An example of plan by GPT 3.5t and solver result by GPT 4o

=== Planner ===

Begin! Describe your plans with rich details. Each Plan should be followed by only one #E.

The 1994–95 U.C. Sampdoria season included a midfielder who was of what nationality?

***Plan: Search for more information about the 1994-95 U.C. Sampdoria season with search tools.***

***#E1 = Wikipedia[1994-95 U.C. Sampdoria season]***

***#E2 = Google[1994-95 U.C. Sampdoria season]***

Plan: Find out the nationality of the midfielder in the 1994-95 U.C. Sampdoria season.

#E3 = LLM[What nationality was the midfielder in the 1994-95 U.C. Sampdoria season? Given context: #E1, #E2]

=== Solver ===

Solve the following task or problem. To assist you, we provide some plans and corresponding evidences that might be helpful. Notice that some of these information contain noise so you should trust them with caution.

The 1994–95 U.C. Sampdoria season included a midfielder who was of what nationality?

Plan: Search for more information about the 1994-95 U.C. Sampdoria season with search tools.

Evidence:

Unione Calcio Sampdoria spent the 1994-95 season mired in the midfield, since its remarkable offensive play from the 1993-94 season failed to occur. Despite this, it came close to another European final, but stumbled at Arsenal following penalties in the semi-finals. Following the end of the season, creative midfielders David Platt, Attilio Lombardo and Vladimir Jugović all left the club, bringing further uncertainty of the future direction of the club.

Plan: Find out the nationality of the midfielder in the 1994-95 U.C. Sampdoria season.

Evidence:

{'type': 'organic_result', 'title': '1994–95 UC Sampdoria season', 'source': 'Wikipedia'}

Now begin to solve the task or problem. Respond with the answer directly with no extra words.

The 1994–95 U.C. Sampdoria season included a midfielder who was of what nationality?

English

Q: The 1994–95 U.C. Sampdoria season included a midfielder who was of what nationality?

Truth: English

Answer: English

***Judgement: CORRECT***
